# Young Adult Resilience for Recovery From Substance Addiction in Assam, India: Lived Experience Insights From a Photo‐Led Interview Study

**DOI:** 10.1002/casp.70022

**Published:** 2024-11-28

**Authors:** Rebecca Graber, Raginie Duara, Sangeeta Goswami, Siobhan Hugh‐Jones, Diptarup Chowdhury, Anna Madill

**Affiliations:** ^1^ Psychology and Counselling University of Chichester Chichester UK; ^2^ School of Psychology University of Leeds Leeds UK; ^3^ MIND India: Institute of Positive Mental Health & Research Guwahati India; ^4^ Lokopriya Gopinath Bordoloi Regional Institute of Mental Health Tezpur India

**Keywords:** peer support, photo‐elicitation, psychological resilience, recovery, substance addiction, visual methods

## Abstract

Substance addiction can be considered a form of social injustice grounded in interactions between individual, family and community‐level risk factors. Although prevention and treatment of substance use disorder is a key target of the United Nations sustainable development goal Good Health and Well‐Being, many low‐and‐middle‐income countries lack a culturally validated approach for its management. We contend that a resilience approach may provide a sound basis from which to develop such an approach in non‐western, low‐resource settings. Hence, the aim of this study is to identify factors supporting resilience for recovery from substance addiction in the lived experience of young adults in Assam, India. We used photo‐led interviews to centre the lived experience of young adult addicts‐in‐recovery (11 men, 5 women; 19–24 years) recruited through two rehabilitation services and their networks. Reflexive thematic analysis of the data produced three clusters of themes: (i) precursors to recovery; (ii) repairing relationships; and, (iii) structuring a life of recovery. Findings are discussed and potential areas for intervention are identified to support a multi‐level, culturally informed, community‐driven approach to recovery from substance addiction.

## 1 Introduction

Prevention and treatment of substance use disorder (SUD) is a target (3.5) of the United Nations sustainable development goal Good Health and Well‐Being. Many factors are associated with the development of substance misuse including genetic and biological, psychological and behavioural, and sociocultural and environmental. There is a global focus on reducing SUDs in young people to mitigate the potential for lifelong disadvantage (Patel et al. 2018) with particular interest in community approaches given resource efficiencies and the inclusion in the recovery process of family and neighbourhood often severely impacted by the drug misuse of members. The present study explores resilience for recovery from substance addiction in the northeastern Indian state of Assam which is responding actively to early and extensive misuse of substances among young people in the region (Pathak et al. 2017; Singh 2022).

Substance addiction is a social justice issue: the onset and treatment of misuse and addiction are grounded in interactions between individual, family and community‐based levels of risk. Substance use is a community‐level risk that shapes the functioning of social relationships and is associated with processes of socioeconomic marginalisation, while those with an addiction face significant stigma and functional barriers to escaping cycles of misuse (Dekkers, De Ruysscher, and Vanderplasschen 2020; Graber 2016; Pisarska et al. 2016). However, much of the research has focused on externalising behaviours (e.g., aggression) rather than, the often less observable, internalising symptoms (e.g., depression) that are frequently comorbid with substance use (Hussong et al. 2011). Moreover, while it is understood that rapid physiological changes during adolescence can affect ‘cognitive reasoning, emotional regulation and risk‐taking behaviour’ (Jiloha 2017, p. 111), the developmental pathways from early childhood to SUD have received comparatively little investigation. This trajectory may be particularly significant in a country such as India where many young people experience tremendous pressure and competition in education and employment in addition to increasing family responsibilities in early adulthood (Agarwal et al. 2013): responsibilities which may conflict with their personal goals (Duara, Hugh‐Jones, and Madill 2021).

In India, young people most vulnerable to addiction include those with a family history of substance use and/or those with emotional and behavioural problems (Mahanta et al. 2016) and vulnerability can lead to active substance misuse through social pressure. In Guwahati, the largest conurbation in Assam, peer influence, ‘fun’, and curiosity are key reasons reported for substance use, with highest rates among middle‐school children (Goswami 2015). Early initiation of drug use can have long‐term detrimental impacts on mental health, physical health and relationships (Jiloha 2017).

Indian government policy around substance misuse aims at treatment and harm reduction (Dalal 2020). Ten Regional Resource and Training Centres assist and mentor more than 400 non‐governmental organisations in addiction services (Avasthi and Ghosh 2019). However, challenges include a focus on managing acute presentation through medical detoxification without long‐term rehabilitation support and poor regulation of private rehabilitation facilities. The Assam State Government is seeking to comprehensively address prevention, treatment and rehabilitation (MSJE 2015). For example, the Scheme of Prevention of Alcoholism and Substance (Drug) Abuse incorporates a range of services for ‘Whole Person Recovery’ to rehabilitate and integrate people recovering from SUD into society through motivation, counselling and after care.

Despite these initiatives, like many low‐and‐middle‐income countries (LMICs), India lacks a culturally validated approach for the treatment and management of SUD. Global peer recovery programmes, such as alcoholics anonymous (AA) and narcotics anonymous (NA), have been somewhat indigenised in India through, for example, incorporating different spiritual beliefs into articulations of a ‘higher power’ (Nimmagadda and Chakradhar 2006). However, a major problem is that even popular psychosocial models of recovery are centred on asset models, identifying a set of formal and informal resources that *individuals* can draw upon to initiate and maintain recovery. That is, focusing on *intra*psychological change rather than supporting conditions at community level. Yet, this approach provides limited scope to examine processes of change operating at ecological levels above the individual and presents an often static view of recovery (Rudzinski et al. 2017). Therefore, we contend that a theoretical framework of psychosocial resilience may provide a sound basis from which to develop culturally appropriate approaches for recovery from SUD.

Psychosocial resilience can be conceptualised in relation to those exposed to significant adversity, as the capacity ‘to navigate their way to the psychological, social, cultural, and physical resources that sustain their well‐being, and their capacity individually and collectively to negotiate for these resources to be provided in culturally meaningful ways’ (Resilience Research Centre 2023, para 3). We are aware of no existing resilience‐based frameworks for *SUD recovery* despite successful application of resilience concepts to recovery from other medical conditions and to *prevention* and *prediction* of substance misuse (Graber et al. 2016; Wu et al. 2018).

A resilience approach extends SUD recovery research in several ways. First, it sensitises researchers to identifying protective mechanisms that enhance recovery at psychological, social and cultural levels, as well as acknowledging biological susceptibility (Graber et al. 2016; Hugh‐Jones et al. 2024; Liebenberg 2020; Rudzinski et al. 2017). Second, adaptation is theorised to develop at different rates along different trajectories at these different levels (Masten 2014). This provides a framework for identifying insights, skills and resources that may be the result of multiple and circuitous pathways (Miller 1996; Sau et al. 2013). For example, even navigating phases of relapse can provide lessons relevant to the ultimate goal of recovery (Madill et al. 2022). Finally, a resilience approach prioritises learning from those with lived experience of journeying through challenging circumstances (Rudzinski et al. 2017).

Innovative methods are required to encourage vulnerable people to participate in research in which they are asked to talk about prior illegal, and possibly traumatising, activities and then to generate rich and useful material (Jiloha 2017; Liebenberg 2020; Sau et al. 2013). We were drawn to visual methods given evidence that they are able to centre the lived experiences of marginalised people, democratise the power dynamic between researcher and participant (Cornell, Mkhize, and Kessi 2019), and are often experienced by participants as intrinsically rewarding (Reavey 2021). Specifically, we used photo‐led interviews because they have demonstrated utility across multiple health contexts and provide impactful material to engage policymakers with research outcomes (Duara, Chowdhury, et al. 2022; Duara, Hugh‐Jones, and Madill 2022; Evans‐Agnew, Rosemberg, and Boutain 2022).

In summary, the aim of this study is to identify mechanisms supporting resilience for recovery from substance addiction in the lived experience of young adults in Assam, India.

## 2 Method

### 2.1 Ethics

Ethical approval was obtained from the Ethics Committee of the LGB Regional Institute of Mental Health, Tezpur, Assam and the Ethics Committee of the School of Psychology, University of Leeds, UK. No ethical concerns arose during the research.

### 2.2 Participants

Participants were recruited through two rehabilitation facilities and their networks in Guwahati, Assam. Candidates for the study were required to be: (i) Indian nationals; (ii) aged 19–24 years; (iii) in recovery from drug and/or alcohol abuse, being at least 1 year substance‐free; and, (iv) feel well enough to take part in the study. Tobacco‐only users were not included given that, in Assam, this is considered unrelated to mental disorder (Pathak et al. 2017). We aimed to recruit more men to reflect the gender ratio in the recovery community of Assam, the final sample comprising 11 men and 4 women. We purposefully recruited for diversity of main substance of addiction (Table 1). Candidates met the Research Fellow (RF) to discuss the study and conditions of consent. If interested in continuing, each was provided guidance on collecting images to bring to interview, for example, avoiding photos of children and of illegal activities.

**TABLE 1:** Participants in order of interview.

Pseudonym	Recruitment source	Gender	Age (yrs)	Main substance	Other substances	Role at time of interview	Interview length (mins)	No. of images
Rishi	PO1	Male	21	Heroin	Alcohol, inhalants, tablets, weed	Service provider	172	20
David	PO2	Male	22	Weed	Alcohol, cough, syrup	Service provider	71	7
Manav	PO1	Male	24	Cocaine	Alcohol, inhalants, weed	Student	138	9
James	PO1	Male	24	Heroin	Alcohol, inhalants, weed	Self‐employed	93	8
Purav	PO1	Male	19	Heroin	Alcohol, inhalants, marijuana, tablets	Service provider	71	33
Rahul	PO1	Male	19	Alcohol	Tablets	Student	55	12
Samar	PO1	Male	22	Cocaine	Weed	Service provider	72	14
Kevin	PO1	Male	21	Heroin	Alcohol, brown sugar, weed	Service provider	122	16
Neo	PO1	Male	24	Heroin	Alcohol, cough syrup, weed	Service provider	134	7
Munu	PO1	Female	24	Alcohol	Brown sugar, weed	Service provider	235	10
Amit	PO1	Male	24	Heroin	Alcohol, tablets	Employed	89	11
Dev	PO1	Male	21	Alcohol	—	Service provider	78	13
Daisy	PO1	Female	23	Heroin	Alcohol, inhalants, tablets, weed	Employed	172	10
Riza	PO1	Female	24	Alcohol	Weed	Unemployed	71	7
Smita	PO1	Female	23	Alcohol	Cannabis, uppers	Self‐employed	138	7

### 2.3 Data Generation

Photo‐led interviews were conducted between April 2019 and October 2020 and were undertaken in the participant's preferred language. Verbal consent was audio‐recorded prior to interview and participants were provided a written copy of the consent form. Consent was reconfirmed when the interview was complete. Interviews commenced by collecting basic demographic information such as age and employment status. The RF then asked: ‘Is there a picture you would like to share first?’ In this way the participant told their story in their own words, embedding the images they had brought into their interview narrative. Prompts were used where appropriate such as, ‘What were your relationships with other people like at this point in your life?’ The interview finished with some reflective questions including, ‘What advice would you give to those trying to tackle alcohol and drug use in young people?’ Audio‐recordings were transcribed in English with portions in Assamese translated and checked. Analysis and data generation progressed concurrently, with notes from the analysis fed back into the interview process. For example, a prompt for sharing experiences of current daily functioning was added when analysis suggested more information would be helpful.

### 2.4 Analytical Procedure

Transcripts were analysed using reflexive thematic analysis, a flexible procedure for identifying patterns of shared meaning in textual data (Braun and Clarke 2006, 2019). Reflexive thematic analysis is, itself, atheoretical and allows researchers to decide the epistemological position in which they wish to operate. The current study takes a contextual constructionist epistemology in which knowledge is considered local, provisional and context dependent (Madill, Jordan, and Shirley 2000). The implication for this study is that we are careful to describe the ways in which our data was generated and analysed and consider the limitations of transferability of our findings in the discussion.

Three researchers analysed the transcripts as they became available, each transcript assigned to a pair. Independently, each researcher made notes on the transcript, then discussed their observations with the other in that pair. One then produced an analytical summary based on identified patterns, concepts and themes which was refined by the other and agreed. The first author then coded each analytical summary inductively with the aim of identifying and describing mechanisms supporting recovery present in participants' lived experience, regularly referring back to the original transcripts, with photographs used illustratively. Resilience was used as a sensitising concept to identify protective mechanisms supporting recovery from substance addiction at individual, family and community levels. This was discussed among the wider research team, with refinements including enhanced consideration of the connections between repairing family relationships and the restructuring of daily life. Finally, the analysis was presented to community partners in Assam consisting of rehabilitation and clinical professionals. They considered the themes clear, usable in recovery programmes, and to have deepened their understanding of their practice.

## 3 Results

The analysis is presented in three clusters of protective mechanisms pulling together themes with similar conceptual meaning: (i) precursors to recovery; (ii) repairing relationships; and, (iii) structuring a life of recovery. The symbol […] is used where a smal section mid‐quote has been omitted for parsimony, short descriptions of people and places, and non‐verbal communication, are provided also in square brackets where this helps understanding, and each quote is indexed with the participant's pseudonym and gender where M = male and F = female. Table 2 maps the ways in which the cluster themes relate to the level of the individual, family and community.

**TABLE 2:** Cluster theme levels of operation and possible intervention.

Cluster theme	Individual	Family	Community
Awareness of services	√	√	√
Understanding addiction	√	√	√
Interrupting physiological processes	√	√	√
Developing self‐love	√		
Sense of belonging	√	√	√
Wanting to put things right	√		
Family adaptation		√	
Emotional literacy	√	√	√
Restructuring time	√	√	√
Envisioning possibilities	√	√	√

### 3.1 Cluster 1: Precursors to Recovery

Before recovery can begin, there are at least three conditions that must be met: (i) awareness of services; (ii) understanding addiction; and, (iii) interrupting physiological processes. Each spans the level of the individual, family and community.

#### 3.1.1 Awareness of Services

Many reported having little to no awareness of services before reaching a crisis point in their addiction. What knowledge they had was often informed by fictional film depictions of asylums and verbal reports of mistreatment: ‘*I thought earlier*, “*What thing is rehab?*” [....] *Some said*, “*In there*, *they tie you up and hit you*”’ (Dev, M). Encounters with professionals, or family members aware of rehabilitation services, could be pivotal, particularly given the centrality of extended family, but was dependent largely on connections and previous experiences: ‘*I told [my school counsellor] that “I want to quit substances.” She happened to know a person* [*…*] *who now has his own rehab. So through this counsellor I visited the rehabilitation’* (Rahul, M). Although awareness of services is necessary, particular barriers for Assamese women are that most services are for men and entering treatment can be very stigmatising. For example, Munu's extended family pressured her parents not to let her go, saying ‘*She is yet to get married. She has a future. Now if a boy comes to see her we cannot say that she has done rehab.* Recovery therefore involves awareness of services, but also the ability and willingness to access those services, which can depend on the family and their perception of implications within the community.

#### 3.1.2 Understanding Addiction

**FIGURE 1:**
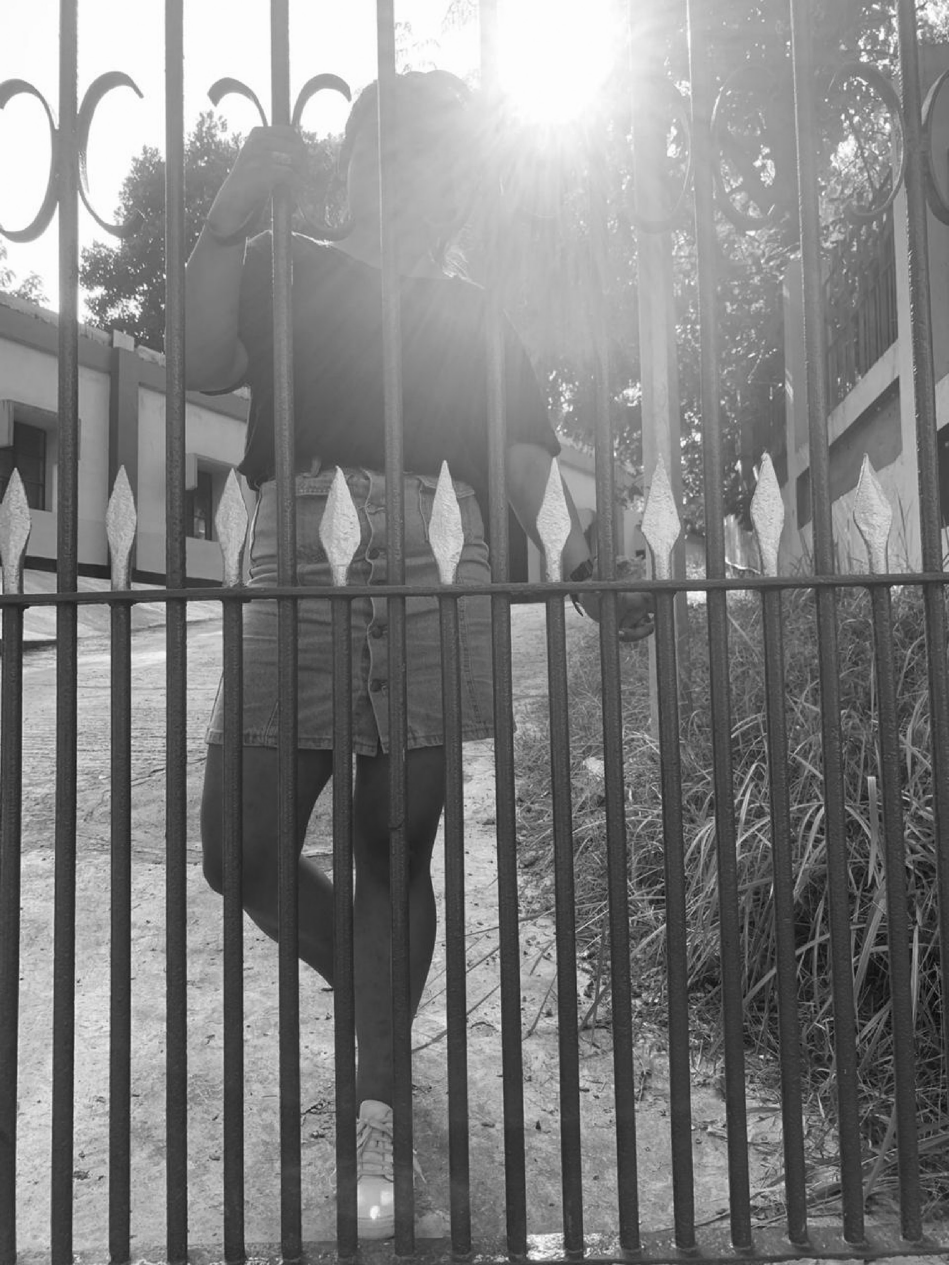
Smita's photograph.

For many participants, some understanding of addiction was essential to start the recovery journey supported by others who are able to see that help is required: ‘*I was very stuck [sniffs] from every side. That means my brother said too “What is that?” Said, “See dear. Try to understand. There is no point doing all these”’* (Smita, F: Figure 1). Moreover, people suffering addiction can experience impulses which lead to antisocial behaviour that contributes to social stigma in that people ‘*directly think like this that, “He is a drug addict. He is a criminal”’* (Neo, M). Hence, it is important that the young person can be viewed by their families and communities separately from the destructive behaviours related to their addiction. However, participants and their families often had little or no knowledge about the cycles of substance use or of withdrawal symptoms to realise that intervention was required: ‘*I told them I want to get myself detoxed. And then we‐ it never really worked and it got worse after that. I was becoming a chronic relapse. After that detox I even told my dad that I am facing this kind of problem and he couldn't make it out only by my looks*' (James, M).

#### 3.1.3 Interrupting Physiological Processes

Interrupting the physiology of addiction, often with pharmacological and other medical support, was for many critical to recovery. However, detoxification was usually experienced as insufficient in and of itself. On the other hand, progress from detoxification to long term, meaningful recovery is was extremely difficult as James explained: *‘When he is high I cannot lecture him because nothing will go into his head. I just can give him the address of the meeting rooms or the rehabilitation centres or I can just tell him that, “Look the way you are leading your life. It's not the way”’*. In fact, increased use of substances prior to entering detoxification rehabilitation was common and a sign that recovery involves addressing a complex constellation of underlying issues: ‘*I did that because* […] *I was feeling bad and the thing I love the most is drugs. I used to love. I still love. When I take it there is no tension in my head and that too I was entering to give it up. First thing is that I would give this up. I won't get it ever. I won't take. Second, I won't ever find my girlfriend’* (Amit, M). Participants and their families often had to learn that psychosocial work would be necessary to support recovery, despite the orientation of services towards detoxification.

### 3.2 Cluster 2: Repairing Relationships

Recovery involves the repairing of relationships. This has four facets: (i) developing self‐love; (ii) sense of belonging; (iii) wanting to put things right; and, (iv) family adaptation. The first three operate at the level of the individual, ‘sense of belonging’ operating also at the level of the family and community, while as the name suggests, ‘family adaptation’ is at the family level.

#### 3.2.1 Developing Self‐Love

**FIGURE 2:**
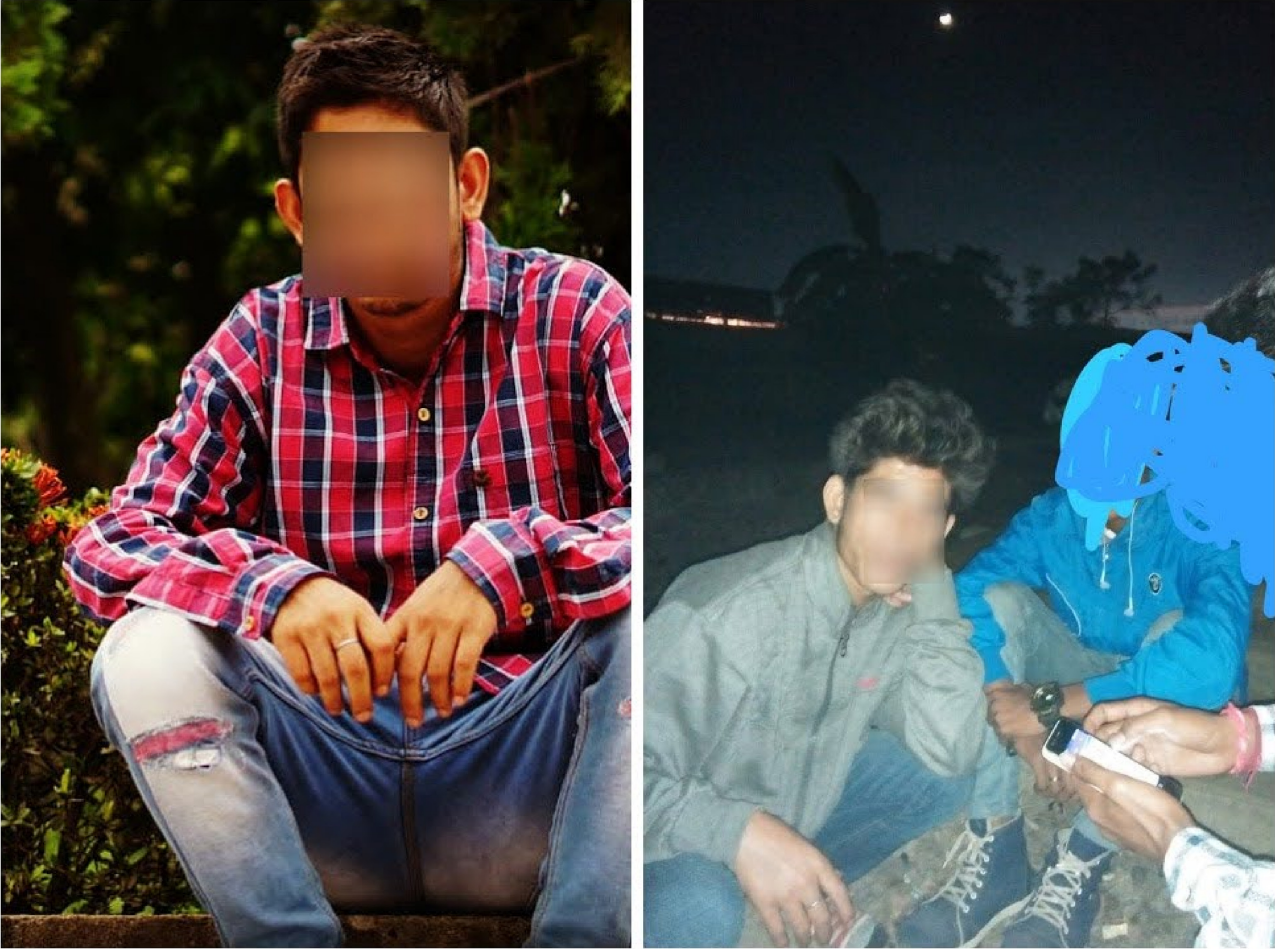
Rishi's photographs.

Self‐love was rarely addressed directly by participants but can be inferred, for example, in the pride taken in improved physical appearance and increasing self‐care as recovery progressed: ‘*today at least if I have to go somewhere I can bathe and* [*…*] *wear good clothes* [*…*] *see myself in mirror* [*…*] *but during my addiction time* [*…*.] *this photo looking how I was. Long hair all messed up’* (Rishi, M: Figure 2). Some participants expressed motivation to protect their bodies from the ‘*dirty pain’* (Dev, M) of addiction. Self‐love could be expressed also through self‐compassion, facilitated by an unconditional acceptance within the peer recovery group: ‘*In sharing* [*…*] *everyone felt this* [*…*] *in this conversation I thought that whatever I did, I did right, whatever I did wrong that also I will take it as good. Without those the right would not have happened’* (Daisy, F).

#### 3.2.2 Sense of Belonging

The sense of belonging provided by peer communities is important in light of the stigma faced by many young adult addicts‐in‐recovery finding new friends, employment, education and, for women, marriage. In particular, a critical feature of rehabilitation and community meetings is the provision of friends in recovery who replace former destructive social networks. Participants were counselled to avoid people who are still using substances: ‘*Today I know that if I go out with my using friends I will definitely get the urge to do it’* (Rishi, M). It can be particularly challenging when extended family used alcohol at gatherings but, with skill, this could be negotiated: ‘*[I] said “No, no. If required I'll leave.” Then they said, “No you don't have to. We will sit somewhere else”’* (David, M). Of particular value to many is mentoring others in recovery, contributing to a social purpose: ‘*I have chosen service* [*…*] *Some people come and tell me that because of you my son has improved. Such things feel good’* (Samar, M).

#### 3.2.3 Wanting to Put Things Right

Participants spoke about coming to recognise the negative impact of their addiction on the people around them. While some focused on romantic partners and friends, family members were overwhelmingly the targets of a desire to put things right: ‘*Till then I started understanding a little how much pain will I cause to mom and dad’* (Kevin, M). However, this process often proceeded unevenly as family and friends struggled to trust in improvements, given cycles of contrition and relapse: ‘*Chacha [uncle] started crying. I felt bad. After I felt bad. That day I didn't smoke. I didn't take any at night. And again I started having it in the next morning’* (David, M). Shame over damaged relationships can trigger relapse although may in the right circumstances also act as a strong motive to seek support with recovery.

#### 3.2.4 Family Adaptation

While family‐initiated interventions were typically ineffectual, they could be the start of joint responsibility for change, with participants eventually experiencing such attempts as acts of love: ‘*otherwise they would not have treated me, worked hard for me’* (Rishi, M). Families needed to (re‐)create a supportive network around love and mutual obligation, for example, Smita describing how responsibility to her brother motivated her to stay clean: ‘*If I have to go for treatment itself for three, four to five months, today who will look after my brother?’* Change might involve (re‐)creating a social network in which love and mutual obligation is placed at the centre of family life: ‘*Now the relationship with sister is good* […] W*hen she couldn't understand something so she would call me* […] *Before she didn't even used to introduce. Nowadays those things are changed. It feels good’* (Manav, M). However, families often needed support to help them adapt to the recovery process and, at times, relationships could be experienced by the young person as too damaged to maintain: ‘*I don't want anything from his side‐ from my father’* (Munu, F).

### 3.3 Cluster 3. Structuring a Life of Recovery

For our participants, recovery requires creating a meaningful life without substances. This involves developing at least three key skills: (i) emotional literacy; (ii) restructuring time; and, (iii) envisioning possibilities. Each spans the level of the individual, family and community.

#### 3.3.1 Emotional Literacy

Although some had the advantage of a stable home and financial situation, many participants described a range of adverse circumstances including domestic violence, family addictions, parental bereavement, academic pressure, bullying, unwanted pregnancy and racism. Whatever their background, many came to understand that they had used substances to escape feelings of guilt, shame and loneliness: ‘*Like normals show, cried and all. Addicts cannot do that. In addicts that stays inside and breaks from within’* (Riza, F). Recovery meant learning new ways to cope and, as David said, ‘*make [my] mind stable’*. This involved attending meetings, conducting emotion inventories, listening to music, meditation, yoga and confiding in others: ‘*When I am very angry at first I think I will go out and have [substance]. I think so okay? I won't lie. It comes. I can't deal with it. My mind is troubled. I dig into my phone* [*…*] *I keep myself amongst people* [*…*] *drive or drive a bike’* (Smita, F).

#### 3.3.2 Restructuring Time

**FIGURE 3:**
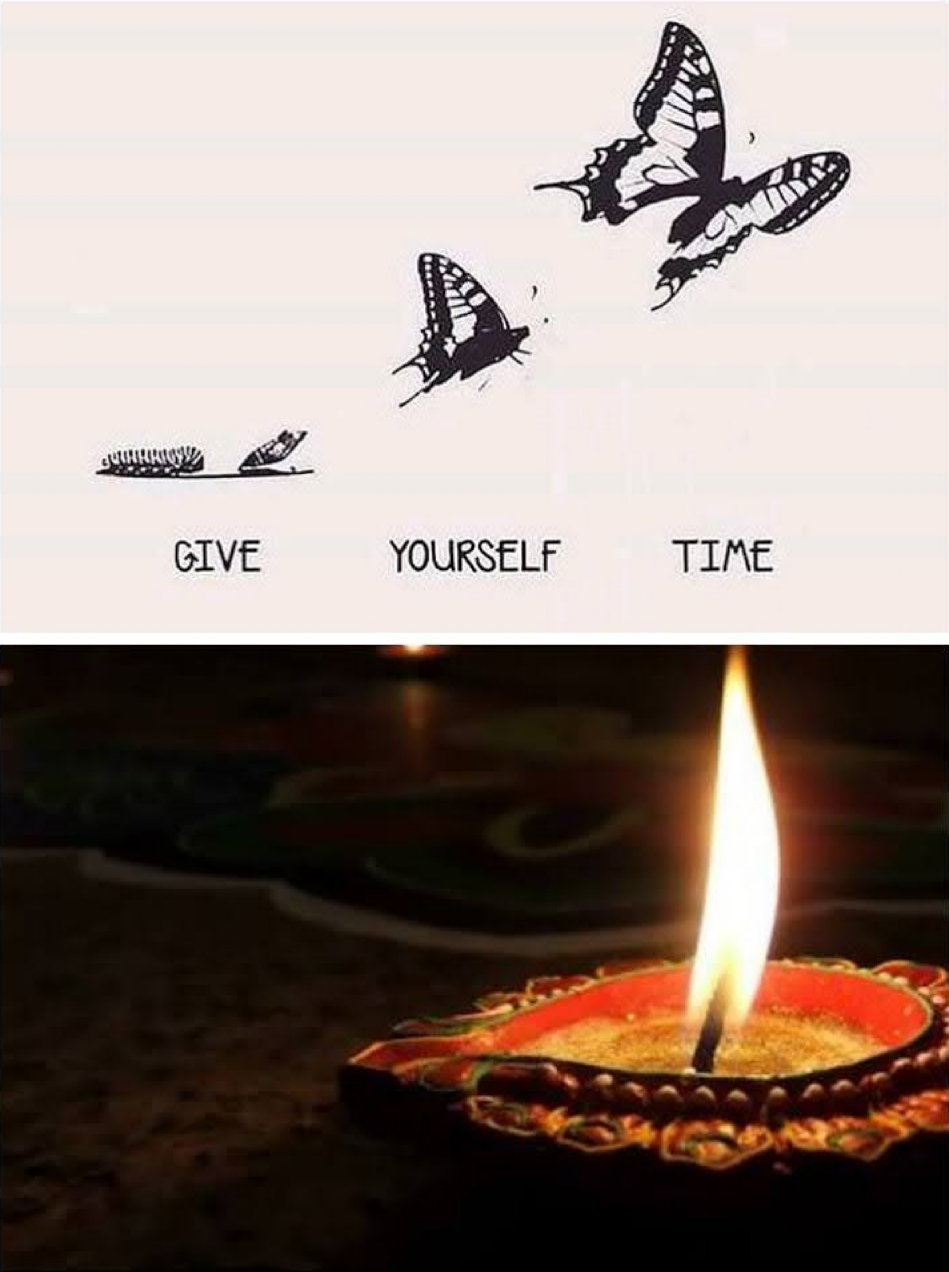
Samar's photograph (above) and Purav's photograph (below).

Addiction disrupts ordinary routines supporting family and community life such as mealtimes, school attendance, basic self‐care and recreational activities such as sports. Rehabilitation facilities helped reinstate a healthy schedule and understanding that recovery requires sustained commitment made manageable through the attitude of ‘*just for today*’ (Daisy, F): ‘*Just think about today. I won't take drugs today. Let's see what will happen tomorrow. So like that, gradually’* (Kevin, M). Learning to combat boredom was a crucial task to avoid relapsing, Rishi noting that *‘if I don't retain a structure in one day then I will get bored doing the same thing again and again*’. Some took the opportunity of ‘giving time’ to support others which contributed to developing patience in their own recovery journey. Hence, in stark contrast to the inertia and chaotic time‐scape of addiction, participants gained a sense of time unfolding in a series of opportunities to actively reconnect with one's own life and one's own body: ‘*I am not like I am just passing time with the things, like just eating, sleeping, keeping away from substances and then it's done’* (Samar, M: Figure 3).

#### 3.3.3 Envisioning Possibilities

Sustainable recovery involves having hope for a purposeful and satisfying future involving interpersonal relationships and goals. However, a key lesson is that, in contrast to the intensity of addiction, a meaningful life may feel mundane and ordinary: ‘*I have to do some work. After working so I'll also want a family. Have to make one’* (Neo, M). Moreover, change can feel impossible, and hope cannot be engineered, as Riza said: ‘*One can explain to a normal but it cannot be explained to an addict*’. Hence, role models are essential, with peers in recovery providing a glimpse of a possible future: ‘*They have stayed clean. People have been respecting them. Plus their relationships that once broke down were getting better again. Then I too saw a hope of life’* (Purav, M: Figure 3). Family acceptance and support can also help the young person leave the past behind, stay grounded in the present and begin to envision a new future: *‘My family comes to know that there is no point of talking about the past so nobody mentions about this* [*…*] *even when there are fights they don't mention about the things from the past’ (*Rahul, M). All this opened up possibilities for the future: *‘When I undertook treatment I understood that there are some beautiful moments in life which I have never seen. Life is actually very beautiful and being in state of intoxication I was making it bad’* (Rishi, M).

## 4 Discussion

The aim of this study is to identify mechanisms supporting resilience for recovery from substance addiction in the lived experience of young adults in Assam, India. Resilience was used as a sensitising concept to identify protective mechanisms supporting recovery at individual, family and community levels. The use of a resilience framework addresses a need for more contextually sensitive approaches to highlight under‐researched processes of recovery from substance misuse, drawing directly from the lived experiences of this socially marginalised group (Graber 2016; Rudzinski et al. 2017). We now discuss each cluster, the implications of our findings, reflect on the strengths and limitations of the study.

### 4.1 Precursors to Recovery

First, it is often the young person's family who are pivotal in initiating the recovery journey through finding out about, securing and funding rehabilitation services. This reflects the importance of extended family in low‐resource settings such as Indian (Duara, Hugh‐Jones, and Madill 2023; Nimmagadda and Chakradhar 2006). Second, it is important for the young person, their family and community organisations to gain some understanding of the addiction process. This can facilitate the young person to accept that a problem exists and to use the support available. Some knowledge of addiction also allows extended family to contextualise past behaviour and to have realistic expectations about recovery. Furthermore, it is important that community organisations, such as schools, recognise the signs of addiction, such as patterns of disengagement, to get young people appropriate support (White 2019). Finally, it is vital to understand that medical detoxification is rarely an effective standalone and that extended psychosocial support is usually required to maintain sobriety (Best and Hamer 2021).

### 4.2 Repairing Relationships

First, self‐love is a focus of many peer recovery programmes and can provide a basis for reaching out to repair damaged relationships (Kissman and Maurer 2002). Aspects of self‐love, such as positive self‐concept, are known to contribute to SUD recovery in non‐western samples, such as in China (Chen, Zeng, and Chen 2020). However, our community partners suggest that promotion of self‐love tends not to be a central feature of Hindu, Muslim or Christian spiritual practices in Assam. In recovery groups, this may be addressed indirectly through practices of connecting with a higher power or non‐judgemental group interactions (Kissman and Maurer 2002) and may explain why our participants implicated the idea of self‐love indirectly, for example through reinvesting in their appearance. It may be that this accommodates aspects of Assamese culture by providing families with a newly presentable young adult who reflects well on them within the community. This is a potentially important re‐contextualisation of the concept of self‐love appropriate to non‐western settings in which the personal change of one individual is highly interwoven with social networks.

Second, young people need to achieve a sense of belonging to replace troubled peer relationships centred on substance misuse. Peer recovery groups frequently meet this need, the sharing of personal experiences creating a sense of connectedness and validation alongside greater understanding of recovery, modification of emotional and behavioural responses, and stigma reduction (Rennick‐Egglestone et al. 2019; Smith‐Merry, Freeman, and Sturdy 2011).

Third, motivation to repair relational damage underpins recovery practices such as making an emotional inventory. Recognising the pain of dysfunctional romantic relationships and the loneliness inherent to social groups structured around substance use, are recognised as recovery motivators alongside the (re)establishment of constructive emotions and behaviours towards people with whom one had a loving bond (Patton and Best 2022). In the most propitious circumstances, the young person's motivation to put things right is encouraged by family adaptation towards relational restitution, the members supporting each other to sustain change (Kelly and Greene 2014).

Finally, kinship networks are central to Assamese culture, meaning that the recovery of one member requires adaptation of the whole family, and it is recognised more generally that family support, as well as vocational rehabilitation, can be critical in the prevention of relapse (Sau et al. 2013). Even so, family adaptation can be difficult and require outside support given the likelihood of addiction having damaged relationships (White 2019). However, as our study demonstrates, if families can be supported to adapt, they can, for example, provide an opportunity for young adult addicts‐in‐recovery to re‐engage caretaking responsibilities which foster meaning, self‐respect and maturity.

### 4.3 Structuring a Life of Recovery

First, our study highlights the value of supporting young addicts‐in‐recovery to develop emotional literacy to cope better with both internalising experiences (e.g., shame) and externalising impulses (e.g., aggression). Interventions to address destructive internalising factors are already a common focus in SUD prevention (Hussong et al. 2011) and could be extended into the recovery period. This is particularly relevant for adolescents and young adults for whom normal developmental opportunities may have been severely interrupted by addiction. Specifically, for these young people, there may be no ‘normal’ to which to return in terms of their social skills, emotional regulation or abstract reasoning (Silvers et al. 2019).

Second, to create a life with meaning beyond addiction, young adults‐in‐recovery need to find a way of restructuring their time. During addiction their time was hyper‐focused on substance‐oriented activities and peer groups, alongside disruption of family, educational and normal recreational routines. Many participants extolled the value of the routines incorporated into rehabilitation facilities as critical to recovery, providing a means to keep busy and a gateway to providing a service to others. Supporting our findings, other research concurs that rehabilitation programme routines can provide a sense of purpose and of control over the environment that helps sustain abstinence (Stokes, Schultz, and Alpaslan 2018).

Finally, the journey to recovery requires envisioning possibilities for a meaningful future without substances. In this regard, it is important to have role models with sustained sobriety, mended relationships and meaningful vocation. Provision of such role models is a key function of peer support in recovery settings (Best and Hamer 2021; Dennis 2003). Envisioning possibilities is also facilitated by a peer recovery community, such as those provided by AA and NA, who can maintain the belief of a hopeful future despite the challenges entailed.

### 4.4 Implications

Our findings suggest that interventions to support young adult resilience for recovery from substance addiction in Assam should be multi‐level, culturally informed and community‐driven. Multi‐level means the incorporation of broader public health interventions as well as targeted, intensive support for individuals and peer groups identified as at particular risk for addiction and their family. Culturally informed means taking the particular strengths and sensitivities of the Assam context, community‐driven through leveraging the motivation and commitment of strong social networks operating in the region. A resilience perspective encourages looking beyond individual assets to more social and structural levers of change, and seeing recovery in terms of an ongoing process instead of a static outcome.

In terms of precursors to recovery, campaigns to enhance awareness of services and understanding of addiction would help combat misinformation about harmful substances, facilitate early identification of problematic substance use and destigmatise help‐seeking (White 2019). Campaigns should be sensitive to local concerns, such as helping families and community leaders to recognise when support from Ayurvedic doctors or gurus may be insufficient. However, although medical professionals play a critical role through providing initial detoxification treatment, this must be followed by long‐term psychosocial support in line with SDG 3.5.1 recommendation that interventions for SUD include psychosocial, rehabilitation and aftercare services (UN 2023). Moreover, effective education needs to engage respectfully with different religions, including the normative use of legal substances such as alcohol, during festivals.

In terms of repairing relationships, drawing inspiration from community in‐reach, extended family networks, religious communities and youth‐based organisations can provide opportunities for genuinely attractive substance‐free belonging (White 2019). Importantly, some consideration is required with regard to including addicts‐in‐recovery in cultural rituals that involve inebriants without prejudicing their sobriety, as has been the focus of recovery support in some Native communities of North America (e.g., Whelshula et al. 2021). Supporting families in their understanding of addiction and in their reintegration of the young addict‐in‐recovery is likely an effective and economical use of resource.

Finally, structuring a life of recovery is often beyond the ability of any one individual given the many influences outside of their control (Liebenberg 2020), and requires an approach of community inclusion (WHO 2021). A resilience framework may be especially useful here. A multi‐tiered approach to emotional literacy through schools and community groups is recommended, with additional support to at‐risk youth. This can act as a preventative measure as well as support the motivation to make amends when in recovery. Service development requires engagement with barriers to use, particularly for women with SUD (Madill et al. 2022). Envisioning possibilities for the future might be promoted through internet‐based interventions which are especially engaging for young people (Jiloha 2017). Finally, economic and cultural interventions providing employment and social opportunities to young adults in‐recovery would facilitate hope for the future in line with SDG target 3.5 to strengthen prevention and treatment of substance abuse—and to promote inclusive growth and productive employment for all (UN 2023).

### 4.5 Strengths and Limitations

Our recruitment processes via two rehabilitation facilities and their networks means that we may have excluded information about recovery processes outside this context. However, research does indicate that recovery without a structured programme of support is difficult (Best and Hamer 2021). Moreover, we did not record caste, religious background or maintenance of sobriety post‐interview all of which may influence transferability of findings. Experiences of SUD recovery include hard to remember periods of time and sometimes traumatic and/or shameful behaviour that are difficult to communicate (Llewellyn‐Beardsley et al. 2019). It is therefore a testament to our photo‐led interview methodology that many participants told us that they had felt supported to tell their own story in their own way. Finally, given our limited sample size, and epistemology position of contextual constructionism, we need to be tentative regarding the transferability of our findings to settings beyond Assam.

## 5 Conclusion

Many LMICs lack a culturally validated approach for the treatment and management of SUD. Addressing a key area of under‐research, this study has identified mechanisms supporting resilience for recovery from substance addiction in the lived experience of young adults in Assam, focusing on what these young people have themselves found meaningful in their recovery. From this we offer recommendations to support recovery from SUD demonstrating that, while intrapsychological assets are necessary, recovery requires also engagement with, and from, families, peer networks and the community.

## Conflicts of Interest

The authors declare no conflicts of interest.

## Data Availability

The data that support the findings of this study are openly available in ReShare at https://reshare.ukdataservice.ac.uk/855418/.
